# Multienvironment Quantitative Trait Loci Analysis for Photosynthate Acquisition, Accumulation, and Remobilization Traits in Common Bean Under Drought Stress

**DOI:** 10.1534/g3.112.002303

**Published:** 2012-05-01

**Authors:** Asrat Asfaw, Matthew W. Blair, Paul C. Struik

**Affiliations:** * Awassa Agricultural Research Centre, Awassa, Ethiopia; † Technology and Agrarian Development Group, Wageningen University, 6706 KN Wageningen, the Netherlands; ‡ Department of Plant Breeding, Emerson Hall, Cornell University, Ithaca, New York 14853; § Centre for Crop Systems Analysis, Wageningen University, 6700 AK Wageningen, the Netherlands

**Keywords:** biomass partitioning, leaf area and chlorophyll content, QTL × environment interaction, nonstructural carbohydrates, photosynthate remobilization and grain yield

## Abstract

Many of the world’s common bean (*Phaseolus vulgaris* L.) growing regions are prone to either intermittent or terminal drought stress, making drought the primary cause of yield loss under farmers’ field conditions. Improved photosynthate acquisition, accumulation, and then remobilization have been observed as important mechanisms for adaptation to drought stress. The objective of this study was to tag quantitative trait loci (QTL) for photosynthate acquisition, accumulation, and remobilization to grain by using a recombinant inbred line population developed from the Mesoamerican intragenepool cross of drought-susceptible DOR364 and drought-tolerant BAT477 grown under eight environments differing in drought stress across two continents: Africa and South America. The recombinant inbred line population expressed quantitative variation and transgressive segregation for 11 traits associated with drought tolerance. QTL were detected by both a mixed multienvironment model and by composite interval mapping for each environment using a linkage map constructed with 165 genetic markers that covered 11 linkage groups of the common bean genome. In the multienvironment, mixed model, nine QTL were detected for 10 drought stress tolerance mechanism traits found on six of the 11 linkage groups. Significant QTL × environment interaction was observed for six of the nine QTL. QTL × environment interaction was of the cross-over type for three of the six significant QTL with contrasting effect of the parental alleles across different environments. In the composite interval mapping, we found 69 QTL in total. The majority of these were found for Palmira (47) or Awassa (18), with fewer in Malawi (4). Phenotypic variation explained by QTL in single environments ranged up to 37%, and the most consistent QTL were for Soil Plant Analysis Development (SPAD) leaf chlorophyll reading and pod partitioning traits. QTL alignment between the two detection methods showed that yield QTL on b08 and stem carbohydrate QTL on b05 were most consistent between the multilocation model and the single environment detection. Our results indicate the relevance of QTL detection in the sites in which bean breeding will be undertaken and the importance of photosynthate accumulation as a trait for common bean drought tolerance.

Common bean (*Phaseolus vulgaris* L.) is one of the major sources of dietary proteins, vitamins, and minerals to millions of resource-poor farmers, particularly in developing countries (Broughton *et al.* 2003). However, yield loss attributable to drought is a major problem for farmers who produce common bean under rain-fed conditions. It is estimated that approximately 60% of common bean production around the globe is affected by drought in any given year, including large areas of Latin America and Africa (Thung and Rao 1999). In Africa, an estimated yield loss of 300,000 MT (metric tons) occurs annually (Wortmann *et al.* 1998), and the yield loss can be up to 80% when severe drought strikes the crop early in crop development (Rao 2001). Despite this, drought is seldom a yearly event, and its effect has both seasonal and spatial variation. On a microregional level, it can occur in different forms either throughout the season, early in the season, at mid-season or near the end of the life cycle of a crop. Thus, drought can have large effects on common bean growth either during early establishment, vegetative expansion, flowering, or grain filling (Rao 2001). Furthermore, climate change will cause greater temperatures and greater evapotranspiration combined with erratic and lower rainfall, which will intensify the problem for small-holder farmers who grow common bean.

Plants usually express differential adaptive strategies to drought stress. These include escape, avoidance, tolerance, and recovery strategies (Levitt 1972), which may not be mutually exclusive but in practice are rarely combined within a single agronomically superior genotype (Ludlow 1989). Instead a range of adaptive responses to drought and mechanisms of drought tolerance are found in different genotypes. Mechanisms that provide drought adaptation in common bean include at a minimum (1) a deep rooting system with an appropriate architecture that increases extraction of soil moisture from a greater soil depth; (2) maximization of water use efficiency for photosynthesis, growth, and development; and (3) greater photosynthate transport to seed under stress through efficient (re-)mobilization (Sponchiado *et al.* 1989; White *et al.* 1994a; Rao 2001; Beebe *et al.* 2008). Finally, phenological plasticity, involving early maturity, drought avoidance, and recovery after drought, is also an important mechanism for plant breeding in short seasons (Acosta-Gallegos and White 1995).

These adaptive strategies for drought stress in common bean are known to be genetically determined for the most part (White 1987, Blair *et al.* 2010, Asfaw and Blair in press); however, full understanding of the underlying genes remains elusive. Plant traits for tolerance to drought have been identified in common bean lines of diverse backgrounds in both the Mesoamerican and Andean genepools (White 1987; Acosta-Gallegos and Kohashi-Shibata 1989, Acosta-Gallego and Adams 1991; Ramirez-Vallejo and Kelly 1998; Muñoz-Perea *et al.* 2006).

Breeding and selection for these traits have resulted in identification of remarkably tolerant parental lines such as BAT477, SEA5, SEA15 and a series of advanced lines in small red, cream stripped, and black seeded commercial grain classes (Singh *et al.* 2001; Teran and Singh 2002; Beebe *et al.* 2008). On-farm testing has shown that drought tolerance is a trait that can contribute to the food security of many resource-poor farmers who live in harsh, low-rainfall environments.

Overall, however, drought tolerance is a physiologically complex trait that must be expressed in terms of increased grain yield under field conditions. In terms of inheritance, drought tolerance is a quantitative complex trait with low heritability for which appropriate selection criteria are largely absent (Schneider *et al.* 1997, Blair *et al.* 2010). In practical terms, selection for drought tolerance is difficult because the drought stress can present itself at different times, with different intensity modified by soil type and fertility (Rao 2001). In the harsh environments in which the majority of smallholder farmers in developing countries grow crops, mechanisms of drought tolerance are difficult to analyze because of the interaction of drought with other stress factors, such as high temperature, low soil fertility, and soil acidity. It is therefore not surprising that drought tolerance is susceptible to genotype × environment (G×E) interaction.

Molecular markers are powerful tools to analyze the genetic control of complex traits such as drought tolerance (Blair *et al.* 2010). Segregation mapping has been used to evaluate quantitative trait loci (QTL) that control multigenic traits such as biomass production and yield partitioning (Collins *et al.* 2008). So far, few QTL analyses for drought tolerance have been reported in common bean, and these have focused on yield components, phenology, and rooting pattern traits (Schneider *et al.* 1997; Blair *et al.* 2010; Asfaw and Blair in press) instead of photosynthate remobilization traits.

The objective of this study, therefore, was to identify QTL associated with photosynthate acquisition, accumulation, and remobilization traits, such as canopy biomass dry weight, biomass partitioning indices, stem and seed total nonstructural carbohydrate content, leaf area index, and leaf chlorophyll content, as well as final yield. This study measured these physiological traits under eight stress and nonstress environments in three different countries and across two continents (Africa and South America) in a recombinant inbred line (RIL) population derived from a drought susceptible × drought tolerant cross.

## Materials and Methods

### Plant material and drought trials under field conditions

An RIL population described in Blair *et al.* (2010) was used for this study. The population consisted of 97 F_5_-derived advanced lines from single seed descent from the cross of DOR364 (drought susceptible) × BAT477 (drought tolerant). DOR364 is a small-red seeded, high-yielding, commercial cultivar developed in Central America that is acceptable in East Africa despite its dark red seed color. BAT477 is a cream-colored, small-seeded breeding line identified by the International Center for Tropical Agriculture (CIAT) as drought tolerant and adapted to various tropical environments. BAT477 was derived from the cross (G3834 × G4493) × (G4792 × G5694) and combines deep rooting ability with greater water uptake efficiency (White *et al.* 1994a, b; Beebe *et al.* 2010). Both parents are indeterminate upright short bush bean of the type II growth habit (Schoonhoven and Pastor-Corrales 1987) and both belong to race Mesoamerica of the Mesoamerican (aka Middle American) gene pool as defined by Singh *et al.* (1991). All RILs plus both parents and one drought tolerant check SEA5 were used in the field experiments.

Field drought phenotyping experiments were conducted at two sites in Ethiopia, one site in Malawi and one site in Colombia. The trials were executed in Colombia in 2007 and in Ethiopia and Malawi in 2009 under the auspices of the Tropical Legumes I project. Drought stressed and nonstressed conditions were applied as separate experiments in each trial site, which created eight environments for QTL analysis. The field site in Colombia (CIAT−Palmira) and its soil and weather conditions are described in Blair *et al.* (2010), whereas the two field sites in Ethiopia were at Awassa and Amaro research farms under the Southern Agricultural Research Institute (SARI). The fourth set of experiments was in Malawi at the Kasinthula research station under the Department of Agricultural Research Services (DARS).

In Ethiopia, Awassa is located at 7°03′N latitude, 38°30′E longitude at an elevation of 1700 m above sea level. The soil at this site is a well-drained sandy loam (Flavisol, FAO classification) with pH 7.0. The yearly average maximum and minimum temperatures of the site are 26.9° and 12.4°, respectively, and annual rainfall is 959 mm on average. Rainfall at this site is divided into 296 mm and 444 mm, respectively, during the short *Belg* rainy season (March−May) and the long *Meher* rainy season (July−October). The Amaro site is located at 5°50′N latitude, 37°55′E longitude at an elevation of 1,426 m above sea level. The soil at this site is a well-drained silt−clay−loam (Eutric nitosols, FAO classification) with pH 6.5. The yearly average maximum and minimum temperatures are 27.6° and 15.2°, respectively, and annual rainfall is 927 mm (412 mm and 294 mm during the *Belg* and *Meher* growing seasons, respectively). In summary for both Colombia and Ethiopia, the rainfall pattern is bimodal at both sites, creating double common bean growing environments in a calendar year. With this in mind, the trials in Ethiopia were planted in *Meher* (from July to October) season at Awassa and in *Belg* (March to June) season at Amaro.

The fourth field site was Kasinthula, Malawi, located at16°0′S latitude, 34°5′E longitude and 70 m above sea level. The yearly average maximum and minimum temperatures of the site are 35.6° and 18.6°, respectively, and annual rainfall is 800 mm on average. The soil surface horizon is sandy loam (United Nations Development Programme 1970), whereas the subsurface horizon is sandy clay loam. Generally the soil is moderately drained with water table at 2.5 m below the ground surface. The average available water holding capacity is 100 mm m^-1^ of soil depth, and the pH is 7.4. The crop was planted off the rainy season, under irrigation in June, using two irrigation regimes: (1) no drought stress—in which the crop was irrigated up to maturity stage, whenever the soil moisture field capacity was depleted by 30%, and (2) drought stress—in which the crop was irrigated up to mid-pod filling stage, whenever the soil moisture field capacity was depleted by 70%, and thereafter the irrigation was cut off completely. In all the experiments except those in Malawi, a 10 × 10 triple lattice treatment design was used. In Malawi the plot sizes were 4 rows of 2 m length by 0.4 m width for Ethiopia sites, two row plots of 4 m length by 0.6 m width for the Colombia site, and single row plots of 2 m length by 0.6 m width for the Malawi site. Recommended packages of agronomic practices were applied at each site.

### Plant trait measurements

A list of the traits evaluated at the different trial sites is presented in Table 1. For quantifying physiological differences in drought tolerance, a number of plant attributes were measured through destructive sampling at mid-pod fill and at physiological maturity. For the plant attributes at mid-pod filling, a row length of 0.5 m (0.2 m^2^ and 0.3 m^2^ area at Awassa and Palmira site, respectively) for each plot was selected, and the plants were cut to the soil surface above the ground and put in a paper bag for processing in the laboratory. Plants were separated into leaves (without petioles), stems, and the remaining (pods and reproductive structures) plant parts. The plant parts were put in separate paper bags and oven dried at 80° for 2 days. After drying of the samples, dry weight of each sample was measured to determine total dry matter production and dry matter distribution into different plant parts (leaf biomass, stem biomass and pod biomass). At harvest, plants within 0.5 m long row (0.2 m^2^ and 0.3 m^2^ area at Awassa and Palmira site, respectively) were cut to the soil surface and oven dried at 80° for 2 days. The oven-dried samples were then separated into plant parts: leaves, stem, pod wall, and seeds, and dry weight measurements were recorded. These data were collected at Awassa and Palmira but not at Amaro and Kasinthula. Physiological traits related with photosynthate accumulation and partitioning included canopy biomass dry weight at mid-pod filling, pod partitioning index, pod harvest index, stem biomass reduction, and harvest index, were measured only in Awassa and Palmira. Canopy biomass dry weight at mid-pod fill was calculated as the sum of dry matter distribution into different plant parts (leaf biomass, stem biomass and pod biomass) at mid-pod and converted into kg ha^−1^. Pod partitioning index was determined as the ratio of dry weight of pods at harvest over dry weight of total biomass at mid-pod fill multiplied by 100. Similarly, pod harvest index was calculated as the ratio of dry weight of seed over dry weight of pod at harvest multiplied by 100. Stem biomass reduction was calculated as stem biomass at mid-pod filling minus stem biomass dry weight at harvest over stem biomass dry weight at mid-pod fill multiplied by 100.

**Table 1:** Plant traits considered for QTL analysis at different sites in three countries (Colombia, Ethiopia, and Malawi) for drought stress and nonstress trials

	Field site			
Traits considered	Awassa	Amaro	Kasinthula	Palmira
Grain yield, kg ha^-1^	√	√	√	√
Canopy biomass dry weight, kg ha^-1^	√			√
Pod harvest index, %	√			√
Pod partitioning index, %	√			√
Stem biomass reduction, %	√			√
Harvest index, %	√			√
Stem TNC, mg g^-1^				√
Seed TNC, mg g^-1^				√
Leaf area index, m^2^ m^-2^				√
SPAD chlorophyll meter reading	√	√*^a^*	√	√
Canopy temperature depression, °C				√

a Evaluation in drought stress environment only. QTL, quantitative trait loci; TNC, total nonstructural carbohydrate content; SPAD, XXX.

In addition, other traits related with drought tolerance that were recorded at mid-pod filling included leaf chlorophyll content, leaf area index, and canopy temperature depression. Leaf chlorophyll content was measured with a SPAD-502 chlorophyll meter (Minolta Camera Co., Ltd, Japan). A SPAD chlorophyll meter reading (SCMR) was recorded on a fully expanded young leaf of one plant for each replication. Leaf area was measured by leaf area meter (LICOR model LI-3000, Lincoln, NE) from plant parts separated as leaves during destructive mid-pod filling plant sampling and converted into leaf area index (m^2^ m^−2^) for statistical analysis. Stem and seed TNC (*i.e.*, total nonstructural carbohydrate content) was determined using NaOH as an extraction medium and anthrone reagent. Absorbance of the solution was measured with a spectrophotometer at 620 nm and TNC concentration was determined by comparison with glucose standards (Kang and Brink 1995). Canopy temperature depression (°C) was measured as the difference in temperature between the leaf canopy and the surrounding air temperature using an infrared thermometer (Telatemp model AG-42D, Telatemp, Fullerton, CA) held at 50 cm from the canopy surface in a 45° angle. Leaf area index, canopy temperature depression, and stem and seed total nonstructural carbohydrate were determined only at the Palmira site, whereas SCMR was evaluated at all four sites. Shoot TNC was measured destructively at mid-pod fill stage, whereas seed TNC was determined at harvest. Data were also recorded at harvest for grain yield (in kg ha^-1^) of all plots in all sites.

### Phenotypic data analysis

Analysis of variance of the variables in both drought-stress and nonstress environments, their genetic parameters such as genotypic and G×E variance components, and phenotypic correlations between grain yield and other variables were conducted using the program Genstat v. 12.1 (Payne *et al.* 2009). A mixed model analysis was used for the analyses of variance, first for each single trait in each single environment and then for G×E following Gilmour *et al.* (1997) using a residual maximum likelihood (*i.e.*, REML) procedure. Block effects were added to the model as random variable when significant for a trait. This was performed to remove the spatial variation within the trial field. Genotypes were considered as fixed to get un-shrunken means (best linear unbiased estimates = BLUEs) for QTL analysis in the best spatial model.

To quantify the severity of drought stress on plant traits, the drought intensity index for each trait was calculated as drought intensity index = 1 – Xds/Xns, where Xds and Xns are the mean experimental trait values of all genotypes grown under drought stress and nonstress, respectively (Fischer and Maurer 1978). For multienvironment analysis, BLUE mean from single environment was used. G×E interactions for the traits measured were assessed using genotype plus genotype × environment interaction (GGE) biplots implemented in Genstat v. 12.1. Genetic correlations for grain yield between trial environments were calculated using factor analytic model of order k = 1 to model the genetic variance−covariance matrix. Broad sense heritability for each trait was estimated as the ratio of genetic variance over genetic variance plus G×E variance obtained from the analysis of variance.

### Molecular mapping and QTL detection

The molecular markers generated for genetic mapping are described in Blair *et al.* (2010). On the basis of these random amplified polymorphic DNA, amplified fragment length polymorphism, and simple sequence repeats markers, a new genetic linkage map was constructed using the software JoinMap v. 4.0 for Windows (http://www.kyazma.nl/index.php/mc.JoinMap/) set to the Haldane 1 mapping function. To create groups, mapping parameters were set to a recombination frequency smaller than 0.15 and a log of odds (LOD) score larger than 5.0. Marker order within a group regression was determined with a mapping algorithm based on the marker order of specific linkage group from previous microsatellite-based common bean genetic maps (Blair *et al.* 2003, 2008). The best marker order of the linkage groups was checked with best plausible positions in maximum likelihood mapping algorithm set to 1000 permutation. Naming of linkage groups was performed by checking for each marker against known marker positions on genetic maps constructed by Blair *et al.* (2003, 2008, 2010). For the final map used in the QTL analysis, a subset of 162 markers was selected in such a way that no two markers were located in the same position so as to improve QTL detection.

QTL and their environmental interaction effects for all the traits were first found by the mixed model−based QTL mapping approach using a single trait and multienvironment option implemented in Genstat 12.1 (Payne *et al.* 2009). For this QTL detection technique, a three-step mixed model analysis as described in Boer *et al.* (2007) was performed using a single interval mapping procedure. In this method based on Bayes theorem and Markov chain methodology (Lander and Green 1987), the QTL effects and positions were estimated on the basis of flanking markers to accommodate uneven coverage of markers along the genome. Hence, the additive genetic predictor or evaluation of positions was determined by marker genotypes predicted at a maximum distance of 10 cM, which resulted in 197 positions tested in the genome. A genome-wide scan for significant QTL expression was performed using the single interval mapping procedure and each predicted marker position fitted as a fixed environment-specific QTL effect while maintaining the best variance-covariance structure based on Schwarz information criterion (Schwarz 1978) set in the Genstat GGE model determined in G×E interaction analysis. The QTL threshold was determined by peak value exceeding a threshold defined for multiple testing by Bonferonni corrections (Lynch and Walsh 1998). The QTL effects were tested by a Wald test (Verbeke and Molenberghs 2000). The amount of variation explained by each QTL was calculated as per Mathews *et al.* (2008). The explained genetic variance (as percentage of the total genetic variance) was calculated as: % explained genetic variance by each QTL = 100 × [1 – (genetic variance in the model with QTL/genetic variance in model without QTL)].

To analyze the frequency of single-environment QTL for each location, a composite interval mapping analysis (Basten *et al.* 2001) was conducted with QTL Cartographer (v. 2.5) based on the data from each location as described in Blair *et al.* (2010). The locations of the mixed-model, multienvironment QTL were compared with the single-environment QTL by map location comparisons on the molecular map drawn to scale for each linkage group.

## Results

### Drought stress under field conditions

The optimum sowing time for common bean was determined for each site and based on this the optimum moment for planting so as to encounter drought stress. In Palmira, the dry season from June to September provided sufficient time for the contrasting irrigated and rain-fed conditions, whereas in Malawi at the lowland site of Kasinthula, the absence of rainfall in the winter season provided similar conditions.

In Ethiopia, the lack of an alternate season and reliable irrigation facilities led us to plant later in the long rainy season to obtain terminal drought stress or near the beginning of the short rainy season for intermittent and terminal drought stress. Near Awassa and for the *Belg* short rainy season, the optimum planting date was at the onset of the rains in March. For the *Meher* long rainy season, we planted in late June to the end of July for full rainfall and later in August for drought stress. Optimum sowing time for common bean at Amaro for *Belg* season was at the onset of the first rainy season in April and for *Meher* season was at the end of August to early September. At this site, *Belg* is the main and relatively better season for common bean production as compared to *Meher*, whereas the reverse holds true for the site at Awassa.

**Figure 1:**
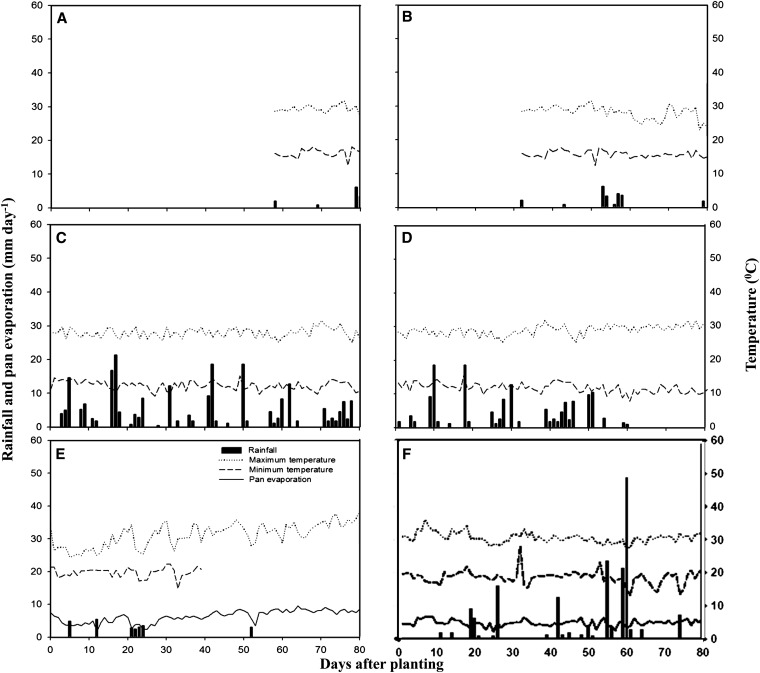
Rainfall distribution, pan evaporation, and maximum and minimum temperature during the crop growth period at different trial locations in Colombia, Ethiopia, and Malawi (for details see *Materials and Methods*). (A) Amaro early planting, (B) Amaro late planting, (C) Awassa early planting, (D) Awassa late planting, (E) Kasinthula, and (F) Palmira.

The weather conditions for each site are shown in Figure 1 (rainfall, pan evaporation, maximum and minimum temperature) during the crop growing period at three locations in the different seasons. The rainfall and temperature data of Amaro, Ethiopia were not available for the full crop growing cycle; however, the average maximum and minimum temperatures, respectively, were 20.2° and 16.0° from 58 to 80 days after planting in the drought nonstress trial [early planting and 26.9° and 15.4° from 32 to 80 days after planting in the terminal drought stress trial (late planting) trial]. The average maximum and minimum temperatures for the other locations were 27.8° and 12.2° in nonstress (early planting) and 28.6° and 11.5° in terminal drought stress (late planting) at Awassa, Ethiopia, 30.9° and 19.4° at Kasinthula, Malawi and 30.6° and 18.6° at Palmira, Colombia. The total rainfall was 235 mm, 149 mm, 25 mm, and 243 mm at Awassa early and late planting, Kasinthula, and Palmira, respectively. The potential pan evaporation was 480 mm at Kasinthula and 431 mm at Palmira.

The weather parameters during the crop growth period indicated that the crop suffered terminal drought stress in late planting trials at Amaro and Awassa, Ethiopia and in the rain-fed trial at Kasinthula, Malawi. By comparison drought stress was intermittent and mainly in early growth crop stages at Palmira, Colombia.

### Yield effects and drought intensity indices

Mean grain yield over the four locations was 62% lower in the stress environment than in the nonstress environments based on drought intensity index calculated from the mean yield of all genotypes under stress *vs.* nonstress. Average grain yields of all genotypes, including parents, RILs, and drought tolerant check were 461, 510, 547, and 988 kg ha^−1^ in the drought stress environments in Amaro, Awassa, Kasinthula, and Palmira, respectively. Meanwhile, in the nonstress environments of the same sites they were and 1207, 2310, 1062, and 2024 kg ha^−1^. The mean grain yield showed 61.8%, 78.0%, 48.5%, and 51.2% reductions due to drought stress in the Amaro, Awassa, Kasinthula, and Palmira sites, respectively. This showed that the effect on grain yield was moderate to severe in drought stress created using early and late planting treatments of Amaro and Awassa compared with the irrigated and rain-fed treatments of Kasinthula and Palmira.

The effect of drought stress was greater on biomass accumulation as compared with photosynthate remobilization. Mean canopy biomass dry weight at mid pod grain filling was reduced by 51% in the stress environments as compared with the nonstress environments. Mean reductions in traits related to photosynthate remobilization to grain were 2% for pod harvest index, 13% for pod partitioning index, 4% for stem biomass reduction, 12% for harvest index, and 10% for stem total nonstructural carbohydrate content. Furthermore, drought stress also caused a 29.5% average reduction in leaf area index and 18% average increase in canopy temperature in the stress environments as compared with the nonstress environments. On the other hand, the mean seed nonstructural carbohydrate was 17% greater under the drought stress environments compared with the nonstress environments, and the SPAD chlorophyll meter readings increased by 4% on average in the same comparison.

### Phenotypic variability of the parents and the derived lines

Significant differences were observed among RILs and between parents for majority of the traits measured in both drought stress and nonstress environments at the four locations and in the eight individual experiments (Table 2). The parental difference was significant for grain yield at all locations except in Kasinthula under nonstress conditions. The drought-tolerant paternal line, BAT477, out-yielded the drought-susceptible maternal line, DOR364, in the majority of the trials except at Amaro and Awassa in the drought stress environments. At Amaro and Awassa drought stress environments, BAT477 was affected by bean stem maggot, which resulted in lower grain yield and lower overall performance. BAT477 was also better in canopy biomass accumulation compared to DOR364 except at Awassa under drought stress. For photosynthate remobilization traits such as pod harvest index, pod partitioning index, stem biomass reduction, and harvest index, the parental differences were not significant except in the Awassa nonstress environment where DOR364 was better for pod harvest index, pod partitioning index, and harvest index and where BAT477 was better for stem biomass reduction.

**Table 2:** Mean values of traits measured in drought-stressed (DS) and nonstressed (NS) conditions at four different locations (Palmira, Colombia in 2007 and Awassa and Amaro, Ethiopia and Kasinthula, Malawi in 2009) for parents DOR364 (P1) and BAT477 (P2) and drought tolerant control check SEA5 along with means and ranges of the RILs from the population DOR364 × BAT477

			Parents			RILs				
Trait	Location	Env.	P1	P2		Mean	Range	*P* value	SEA5	Avg. SED
Grain yield, kg ha^-1^	Amaro (Eth)	DS	646	146		460	77-947	<0.001	1001	79.7
		NS	1849	2338		1202	192-2412	<0.001	1433	481.1
	Awassa (Eth)	DS	522	476		503	219-1003	<0.001	391	77.1
		NS	2118	2908		2361	1150-3622	<0.001	1852	261.0
	Kasinthula (ML)	DS	510	661		551	268-855	0.04	457	124.9
		NS	1344	1111		1068	517-1583	0.074	964	ns
	Palmira (Col)	DS	956	1126		986	709-1340	0.000	911	273
		NS	2075	2171		2029	1595-2556	0.013	2010	446
Canopy biomass, kg ha^-1^	Awassa (Eth)	DS	3015	1958		2479	1062-4270	<0.001	2915	487
		NS	2851	4480		4243	2328-6783	<0.001	2895	546
	Palmira (Col)	DS	2163	2517		2223	1441-3269	0.008	2082	408
		NS	6180	6445		5362	4166-6930	0.273	5491	ns
Pod harvest index, %	Awassa (Eth)	DS	72.9	72.5		72.2	57.0-81.8	<0.001	68.4	3.6
		NS	76.1	71.9		70.7	58.3-80.8	<0.001	65.8	3.5
	Palmira (Col)	DS	76.1	76.2		75.6	68.8-79.3	0.002	77.3	2.0
		NS	79.3	80.0		79.9	76.3-83.1	<0.001	80.1	1.1
Pod partitioning index, %	Awassa (Eth)	DS	33.5	35.6		34.7	15.2-84.3	<0.001	37.0	8.6
		NS	97.4	74.7		66.8	29.7-97.7	<0.001	90.1	10.7
	Palmira (Col)	DS	56.7	54.7		67.5	37.4-87.7	0.335	71.1	ns
		NS	38.8	60.1		50.9	32.7-76.7	0.551	51.5	ns
Stem biomass reduction, %	Awassa (Eth)	DS	31.9	26.5		31.5	3.82-60.4	0.125	20.9	ns
		NS	21.7	33.6		43.5	6.9-70.5	<0.001	11.4	15.1
	Palmira (Col)	DS	46.1	46.6		43.9	20.1-67.6	0.855	45.5	ns
		NS	34.5	31.1		34.9	11.0-57.1	0.447	19.2	ns
Harvest index, %	Awassa (Eth)	DS	24.3	25.9		24.9	10.3-59.3	<0.001	25.4	6.5
		NS	73.9	53.6		47.5	18.0-73.6	<0.001	59.4	8.3
	Palmira (Col)	DS	43.6	42.2		52.5	28.7-94.4	0.396	56.9	ns
		NS	31.1	48.1		40.9	25.4-61.8	0.497	40.6	ns
Stem TNC, mg g^-1^	Palmira (Col)	DS	227	131		199	125-278	0.756	206	ns
		NS	229	229		220	125-318	0.308	220	ns
Seed TNC, mg g^-1^	Palmira (Col)	DS	261	332		358	272-451	0.101	501	61.9
		NS	333	261		310	244-398	0.120	320	ns
SPAD chlorophyll meter reading, SCMR	Amaro (Eth)	DS	24.7	24.6		24.3	18.9-30.1	0.078	26.4	ns
	Awassa (Eth)	DS	24.3	24.0		22.2	16.1-28.7	<0.001	22.5	2.5
		NS	23.3	18.3		20.3	14.6-26.5	<0.001	20.8	2.7
	Kasinthula (Mlw)	DS	41.0	42.5		40.9	34.7-47.7	0.417	44.5	ns
		NS	37.9	37.3		39.3	32.5-45.9	0.690	46.6	3.3
	Palmira (Col)	DS	41.9	38.1		41.9	34.8-49.6	<0.001	39.5	3.0
		NS	31.6	34.6		32.5	20.5-46.2	<0.001	37.7	4.8
Leaf area index, m^2^m^-2^	Palmira (Col)	DS	1.63	1.83		1.70	1.10-2.41	<0.001	1.35	0.29
		NS	2.88	2.90		2.38	1.34-3.41	<0.001	1.98	0.42
Canopy temperature depression, °C	Palmira (Col)	DS	3.54	4.14		3.35	1.11-6.36	0.005	4.70	1.12
		NS	3.82	4.60		4.11	1.62-6.26	0.347	4.94	ns

Mean values are of three replications in each experiment. *P* value indicates level of significance for genotypic difference among RILs for each trait. Average standard error of the difference (AvSED) indicates the genotypic difference to declare significance among RILs, parents, and the check genotype SEA5, advanced line from CIAT. RIL, recombinant inbred line; Env, environment; Eth, Ethiopia; Col, Colombia; ML, Malawi; TNC, total nonstructural carbohydrate content; P1, DOR364; P2, BAT477; SCMR, SPAD chlorophyll meter reading; ns, nonsignifiant.

For stem nonstructural carbohydrates, no differences were found both for the parents and the derived lines in any of the environments, whereas for seed nonstructural carbohydrates BAT477 was better than DOR364 under drought stress in Palmira, the only site at which these traits were measured. It would have been interesting to observe the results of these two traits in the other sites, but the laboratory for measuring nonstructural carbohydrates was specific to the Palmira site and seed and stem shipments could not be arranged because of quarantine requirements.

For other traits, namely the SPAD chlorophyll meter readings, the results were generally greater in drought stress environments than in nonstress environments for both parents and RILs, whereas leaf area index was higher in nonstress environments than in drought stress environments. Using a temperature gun, BAT477 was found to be excellent, in keeping the canopy temperature cooler as compared with DOR364 in both drought stress and nonstress environments. The drought tolerant control SEA5 was inconsistent in performance, sometimes being found to be better or worse than the parents for many of the traits measured across locations and stress levels.

**Figure 2:**
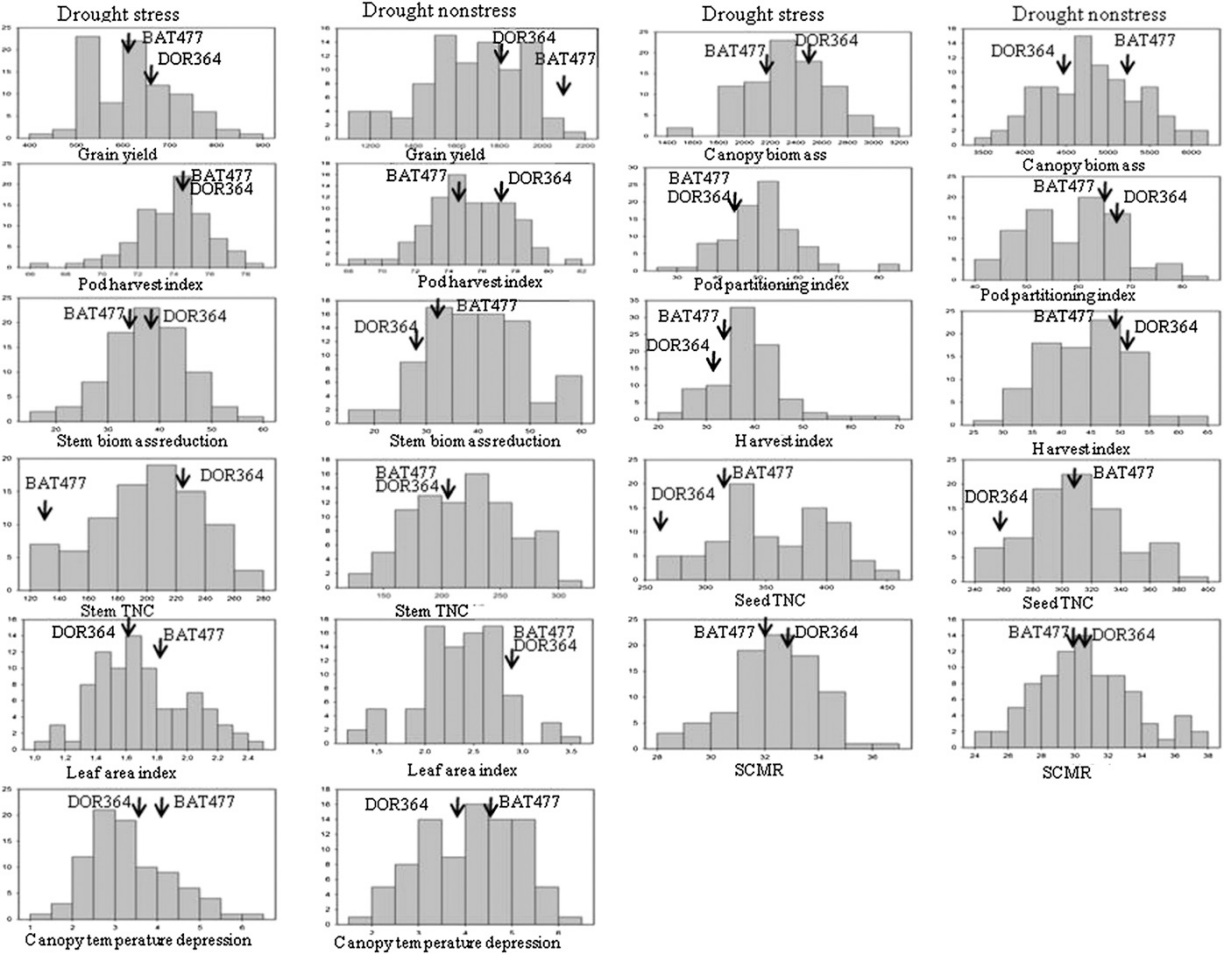
Frequency distributions for mean phenotypic values over locations among the RILs of the DOR364 × BAT477 population grown under stress and nonstress environments. Arrows indicate the values of the parents.

The RIL population distributions were continuous for all traits, suggesting quantitative inheritance in all cases and both in drought stress and nonstress environments (Figure 2). However, some skewing in distribution was observed for grain yield at Awassa in both drought stress and non-stress environments. Kurtosis was also significant for some partitioning traits (data not shown), mainly pod-partitioning index and harvest index at Awassa under drought stress. Some transgressive segregation was observed among the RILs for all the traits measured at each location and in each environment. This transgressive segregation was found both in positive and negative directions (Table 2). At each trial site and in each environment, several RILs were better or worse than the drought-tolerant paternal line BAT477 or drought-susceptible maternal line DOR364 for all the traits measured by this study.

### G×E interaction, heritability, and correlation

The genotypic (G) and G×E variance component and broad-sense heritability for all traits measured are presented in Table 3. The G×E variance was greater than genotypic variance for all the traits except leaf area index and SCMR confirming highly specific environmental effects on the expression of these drought tolerance traits. Genetic variance was equal to G×E variance for leaf area index, whereas it was a little greater than the interaction effect for SPAD chlorophyll meter readings. This finding indicated the relatively high across environment repeatability of these two traits as compared with other traits measured in the study. The broad-sense heritability was low to medium in value for all the traits measured, being especially low for pod-partitioning index. The highest average heritabilities considering all sites were for leaf area index (0.51) followed by seed TNC (0.50), stem TNC (0.45), SPAD chlorophyll meter readings (0.43), and canopy temperature depression (0.42). Medium-to-low heritabilities (0.35−0.39) were observed for pod harvest index, grain yield, canopy biomass dry weight at mid-pod fill, stem biomass reduction, and harvest index.

**Table 3:** Estimates of genotypic (G) and genotype × environment (G×E) variance components, broad sense heritability (h^2^_b_), and phenotypic correlation coefficients (r_p_) of physiological traits with final grain yield under drought stress and nonstress trials in four locations across three countries (Colombia, Ethiopia, and Malawi) for the DOR364 × BAT477 population

				r_p_ with Grain Yield Under							
	Variance Component			Drought Stress					Nonstress		
Traits	G	G×E	h^2^_b_	PAL	AW	AM	KAS		PAL	AW	KAS
Grain yield, kg ha^-1^	2754	4669	0.37	1.00	1.00	1.00	1.00		1.00	1.00	1.00
Canopy biomass, kg ha^-1^	20979	38058	0.36	0.41^***^	0.20^*^				0.25^**^	0.49^***^	
Pod harvest index, %	0.515	0.792	0.39	0.25^**^	−0.21^*^				0.11	0.20^*^	
Pod partitioning index, %	2.61	12.0	0.18	0.15	0.30^***^				−0.02	−0.18^*^	
Stem biomass reduction, %	6.5	11.9	0.35	0.03	0.08				0.06	0.06	
Harvest index, %	4.62	8.65	0.35	0.10	0.28^**^				0.00	−0.11	
Stem TNC, mg g^-1^	165	203	0.45	−0.02					−0.02		
Seed TNC, mg g^-1^	175	246	0.50	−0.09					0.03		
Leaf area index, m^2^ m^-2^	0.0154	0.0154	0.51	0.46^***^					0.19^*^		
SPAD chlorophyll meter reading	0.550	0.539	0.43	0.20^*^	−0.02	0.13	−0.01		−0.21^*^	−0.37^***^	0.02
Canopy temperature depression, °C	0.1046	0.1379	0.42	−0.05					0.11		

Significant at ^*^P < 0.05, ^**^P < 0.01, and ^***^P < 0.001 (one-tailed). PAL, Palmira; AW, Awassa; AM, Amaro; KAS, Kasinthula, TNC, total nonstructural carbohydrate content.

**Figure 3:**
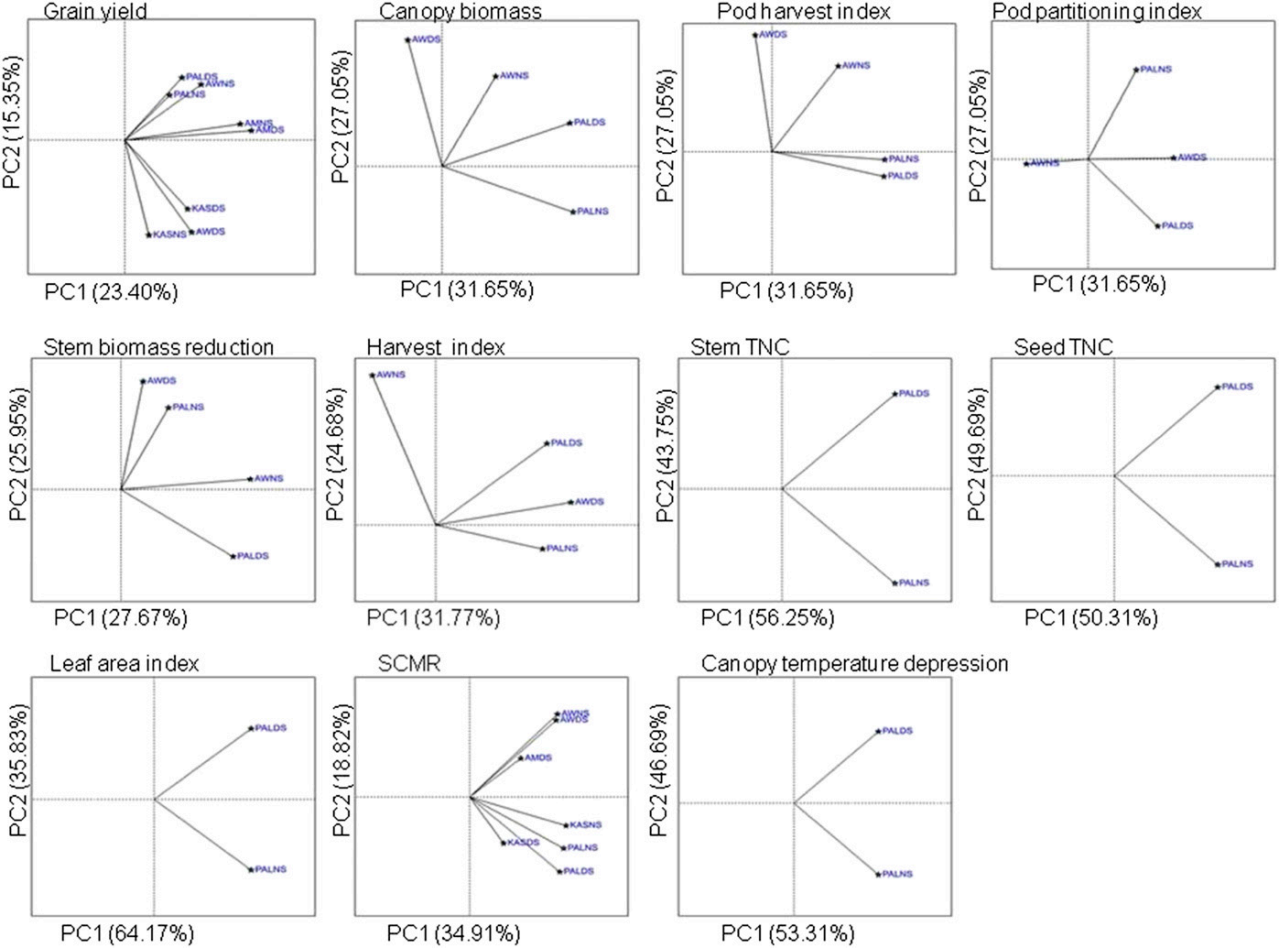
Biplots indicating pattern of G×E interaction for different traits measured in this study. Trait name indicated on top of respective biplot.

Figure 3 shows the patterns of G×E interaction in the experiment. Using a set of biplot displays, we found that the lengths of the vectors connecting the environment to the origin corresponded to the amount of genetic variation expressed in that environment. Meanwhile, in the same biplots the cosine of the angle between environmental vectors approximated the correlation between environments with respect to the G×E interaction. Acute angles between two environments represented high positive correlations, whereas wide obtuse angles (>90°) between two environments indicated their dissimilarity. G×E interaction effects for all traits measured were mainly caused by contrasting effects of each trial environment. For example, for grain yield in the Palmira nonstress environment, relatively little G×E interaction was observed compared with the drought stress environment, perhaps due the deep soils and high water table at this site. In contrast, strong environmental dissimilarities were observed between Palmira drought stress and nonstress environments compared with Awassa nonstress and Kasinthula drought stress environments. Similarly, nonstress environments were contrasting with the Awassa drought stress environment for grain yield; and finally, Amaro drought stress and nonstress environments tended to be closely correlated.

In general, low genetic correlations were observed both within African trial sites and between Africa and Colombia trial sites for grain yield performance (Table 4). The greatest correlation was between environments at the Amaro site (*r* = 0.41, *P* < 0.001); however, correlations between drought stress and nonstress environments were not statistically significant at the other three sites (*r* = 0.01−0.09). Some significant correlations were observed between Awassa and Amaro for nonstress environments (*r* = 0.19, *P* < 0.05) and drought (*r* = 0.20, *P* < 0.05) environments. Interestingly grain yield in the Awassa nonstress environment was correlated with the Amaro drought stress environment (*r* = 0.26, *P* < 0.01). The wide obtuse angles between Awassa drought stress and Palmira non-stress environments for canopy biomass dry weight at mid-pod fill, for pod harvest index and for harvest index indicated crossover type G×E interaction. Patterns of G×E interaction for pod-portioning indices reflected the contrasting effects of drought stress and nonstress environments on this trait. For SPAD chlorophyll meter reading, Amaro and Kasinthula drought stress environments contributed low G×E interaction variance.

**Table 4:** Genetic correlations between drought stress (DS) and nonstress (NS) trial environments in four locations across three countries (Colombia, Ethiopia, and Malawi) using a factor analytic model of order k = 1 selected for modeling the residual genetic variance-covariance matrix for grain yield

Trial	AMDS	AMNS	AWDS	AWNS	PALDS	PALNS	KASDS
AMNS	0.41^***^						
AWDS	0.20^*^	0.15					
AWNS	0.26^**^	0.19^*^	0.09				
PALDS	0.18^*^	0.13	0.06	0.08			
PALNS	0.12	0.09	0.04	0.05	0.04		
KASDS	0.19^*^	0.14	0.07	0.09	0.06	0.04	
KASNS	0.04	0.03	0.02	0.02	0.01	0.01	0.01

Significant at ^*^P < 0.05, ^**^P < 0.01, and ^***^P < 0.001 (one-tailed). AMDS, Amaro drought stress; AMNS, Amaro nonstress; AWDS, Awassa drought stress; AWNS, Awassa nonstress; PALDS, Palmira drought stress; PALNS, Palmira nonstress; KASDS, Kasinthula drought stress; KASNS, Kasinthula nonstress.

The correlation values between traits and grain yield are shown in the last columns of Table 3 for both drought and nonstress environments. Positive and significant associations were observed between grain yield and biomass dry weight at mid-pod filling across locations and drought stress and non-stress environments. Under drought stress environments, the correlations with grain yield were positive (although not always significant) for photosynthate partitioning, pod harvest index, stem biomass reduction, and harvest index across locations, except for the negative and significant correlation for pod harvest index in Awassa. Pod harvest index and stem biomass reduction had positive correlations with grain yield under nonstress environments, whereas they were negative or zero for pod partitioning index and harvest index under the same set of conditions. The relationship between grain yield with stem or seed TNC were nonsignificant. However, for grain yield and leaf area index they were positive and significant both under the drought stress and the non-stress environment (only assessed in Palmira).

The relationship between grain yield and SPAD chlorophyll meter reading was inconsistent across locations and environments. It was negative under non-stress conditions in Awassa (*r* = −0.38, *P* < 0.001) and Palmira (*r* = −0.21, *P* < 0.05), whereas it was positive under drought stress conditions in Amaro (*r* = 0.13, *P* = 0.102) and Palmira (*r* = 0.20, *P* < 0.05). The correlation values between grain yield and SPAD chlorophyll meter reading were not significant in Kasinthula under both drought stress and nonstress environments and in Awassa under drought stress. Grain yield was weakly correlated with stem and seed TNC, and canopy temperature-depression both under stress and nonstress environments.

### QTL mapping with multienvironment mixed-model and single-environment composite interval mapping analyses

Three types of marker systems, namely amplified fragment length polymorphism, random amplified polymorphic DNA, and simple sequence repeats/microsatellites, were used to generate the linkage map for QTL detection. The DNA polymorphism level with the molecular markers used in this Mesoamerican intra-genepool cross was low to moderate depending on the marker type as described in greater detail in Blair *et al.* (2010). As distinct from that study, the linkage map presented here was constructed with a regression mapping algorithm using a total of 165 of the 205 polymorphic markers evaluated. This linkage map covered all 11 linkage groups of the bean genome, with a total genetic distance of 798.6 cM, and had an average length *per* linkage group of 71.8 cM, with an average distance between markers within a linkage group of 14.7 cM. The advantage of this genetic map for QTL analysis was that mapping positions were represented by nonconflicting and nonoverlapping markers such that a genome scan procedure was appropriate for all traits.

**Figure 4:**
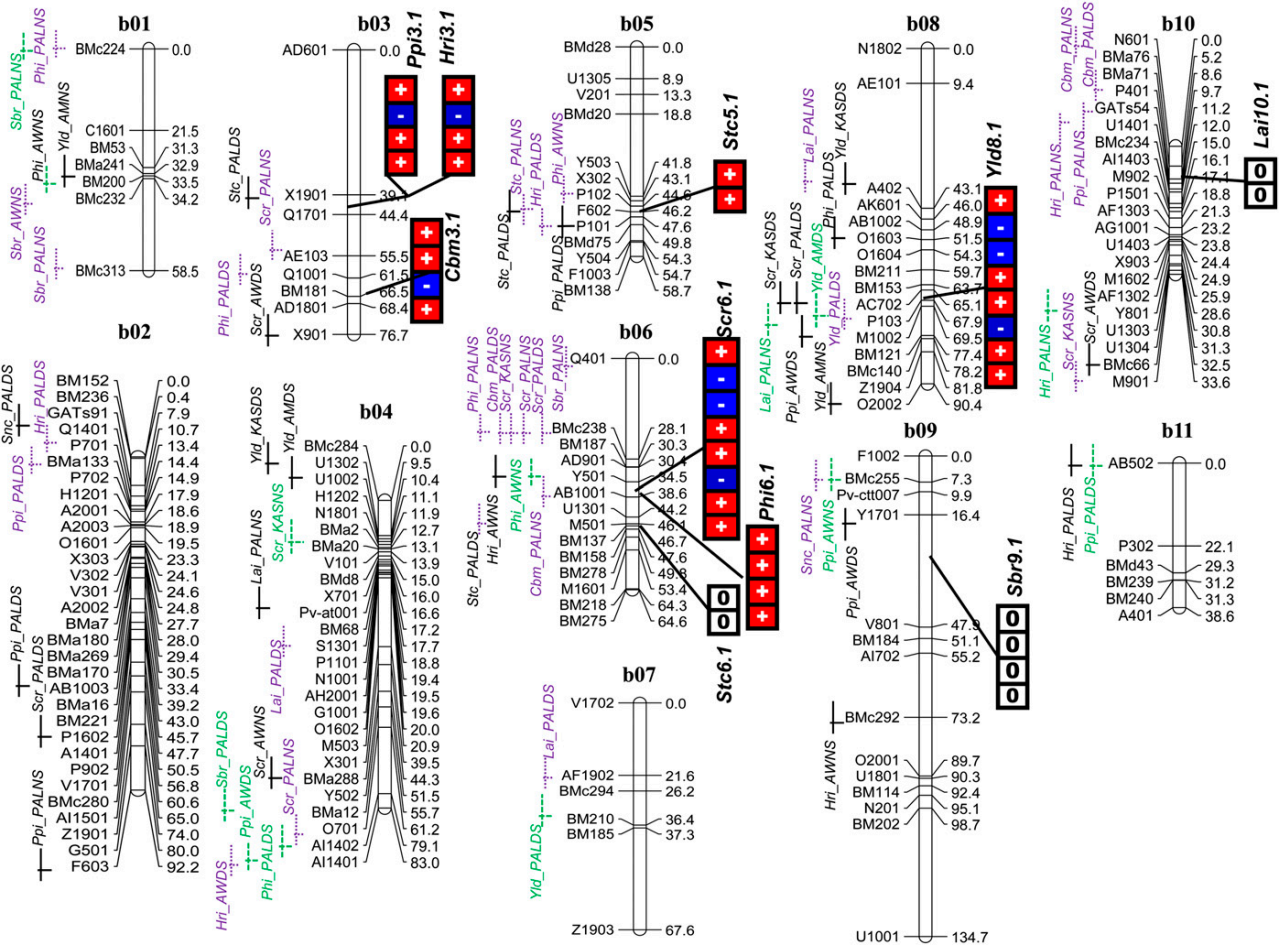
Distribution of QTL for photosynthate acquisition, accumulation, and partitioning traits detected in multienvironment mixed model (right side of the linkage group) and site-specific composite interval mapping (left side of the linkage group) on the 11 linkage groups of the DOR364 × BAT477 genetic map (refer to Table 5 and 6 for QTL names and to Blair *et al.* (2010) for base genetic map). Vertical bar in right side of the linkage group with connectors to the corresponding positions on the linkage group represented each QTL with mixed model. Colocalizing QTL in mixed model indicated by connector pointing more than one trait. The blocks in vertical bar indicate the effect of the QTL in each environments (from top to bottom the environments are: AMDS, AMNS, AWDS, AWNS, KASDS, KASNS, PALDS, and PALNS for grain yield; AMDS, AWDS, AWNS, KASDS, KASNS, PALDS, and PALNS for SPAD chlorophyll meter reading; AWDS, AWNS, PALDS, and PALNS for biomass accumulation and partitioning traits; and PALDS and PALNS for stem total nonstructural carbohydrate and leaf area index (refer to Table 4 for environment abbreviations). The environment-specific effect of the parental marker alleles are indicated by either a ‘+’ sign (red background) or a ‘−’ sign (blue background) in the vertical bars. A ‘+’ sign or red background represents the drought-tolerant paternal line BAT477 marker allele increasing the traits value, whereas a ‘−’ sign or blue background represents the drought tolerance from the susceptible maternal DOR364 marker allele. Main effects are indicated by ‘0’ (white background). The QTL detected with composite interval mapping are indicated at the left side of each linkage group with a black solid line, a dashed line or a dotted line indicating significance at threshold LOD scores of 2.0, 2.5, and 3.0, respectively.

With the multienvironment, mixed-model genome scan, a total of nine significant QTL were identified. These were associated with yield, canopy biomass, and photosynthate accumulation and partitioning related traits (Table 5). QTL were detected on 6 of the 11 linkage groups, including b03, b05, b06, b08, b09, and b10 (Figure 4). No significant QTL were detected for seed TNC and canopy temperature depression; however, QTL were identified for stem TNC and for SPAD chlorophyll meter readings. The significant QTL were named by combining a three-letter code for the trait with the linkage group and the order of the QTL for the given trait on each linkage group. The QTL for canopy biomass dry weight at mid-pod filling, pod harvest index, stem TNC, and SPAD meter reading were significant with threshold LOD scores of 3.125 whereas QTL for grain yield, stem biomass reduction, harvest index, and leaf area index were significant with LOD scores of 2.75. The QTL for grain yield on b08, canopy biomass dry weight at mid-pod fill on b03, for pod harvest index on b06, for pod partitioning harvest index on b03, for stem TNC on b05, and for SPAD chlorophyll meter reading on b06 showed inconsistent effects across environments whereas other QTL did not. The QTL × environment interaction effects for grain yield, canopy biomass dry weight, and pod partitioning index were of crossover types, whereas the interaction was noncrossover type for pod harvest index, stem TNC on b05, and SPAD chlorophyll meter reading. The QTL for stem TNC on b06 was consistent across environments. The crossover QTL × environment interaction effects in the study population were attributed to the contrasting effect of the parental alleles across different environments.

**Table 5:** Significant QTL and the percentage of genetic variance explained by the full QTL model for photosynthate acquisition, accumulation, and partitioning traits in the DOR364 × BAT477 mapping population grown under drought stress and nonstress conditions at four different locations in three countries (Colombia, Ethiopia, and Malawi) using multienvironment mixed model genome scan

							% of Genetic Variance*^b^*	
Trait	QTL*^a^*	LG	Marker	LOD	Wald	Pr_wald_	Stress	Nonstress
Yield	*Yld*8.1	8	P103	2.83	3.03	0.003	19.45	14.45
Canopy biomass	*Cbm*3.1	3	AD1801	4.14	3.37	0.011	0.70	5.35
Pod harvest index	*Phi*6.1	6	Y501	3.30	4.92	0.001	0.00	6.45
Pod partitioning index	*Ppi*3.1	3	Q1701	3.14	3.28	0.013	0.95	10.00
Stem biomass reduction	*Sbr*9.1	9	Y1701	2.88	8.42	0.004	2.15	4.20
Harvest index	*Hri*3.1	3	Q1701	2.85	3.03	0.019	0.65	4.55
Stem TNC	*Stc*5.1	5	F601	4.77	11.72	0.000	1.20	17.60
	*Stc*6.1	6	M501	3.68	17.35	0.000	11.60	3.60
SPAD chlorophyll meter reading	*Scr*6.1	6	BMc238	10.79	11.39	0.000	19.68	42.23
Leaf area index	*Lai*10.1	10	N601	2.76	11.52	0.001	0.00	3.40

QTL, quantitative trait loci; LG, linkage group; LOD, log of odds; Pr_wald_ = Wald probability; TNC, total nonstructural carbohydrate content; SPAD, XXX.

a QTL name based on association with yield (*Yld*), canopy biomass (*Cbm*), pod harvest index (*Phi*), Pod partitioning index (*Ppi*), stem biomass reduction (*Sbr*), harvest index (*Hri*), stem total nonstructural carbohydrate (*Stc*), SPAD chlorophyll meter reading (*Scr*), and leaf area index (*Lai*). Numbers indicate chromosome association (first number) and order of identification (second number after decimal point).

b The percentage of variance explained for each QTL under the full QTL model drought stress and nonstress conditions was calculated as average across the sites for the trait in respective drought stress and nonstress environments.

Figure 4 shows the results of the test for the specific effect of each QTL at each test environment. For grain yield, the alleles from the drought-resistant parent BAT477 had an increasing effect at Amaro drought stress, Awassa nonstress, Kasinthula drought stress, Palmira drought stress, and nonstress conditions, but these BAT477 alleles had a decreasing effect at Amaro nonstress, Awassa drought stress, and Kasinthula nonstress conditions. For canopy biomass dry weight at mid-pod filling the alleles from BAT477 had an increasing effect at Awassa, both under drought stress and nonstress conditions and at Palmira under nonstress conditions and a decreasing effect at Palmira under drought stress. The colocalizing QTL for biomass partitioning traits, namely pod partitioning and harvest index QTL on b03, showed a positive effect from the BAT477 alleles at Awassa drought stress and at Palmira under drought and nonstress conditions. The QTL × environment interaction effects for pod harvest index and SPAD chlorophyll meter reading were attributed to differential expression of the BAT477 paternal alleles, which increased the trait value of pod harvest index and decreased the trait value for SPAD chlorophyll meter reading across test environments. The QTL for stem biomass reduction, stem TNC on b06 and leaf area index were main effect QTL exclusively.

The genetic variance accounted for by the final QTL model for each trait was as high as 19.7% for the SPAD chlorophyll meter reading QTL on b06 (*Scr*6.1) and 19.5% for grain yield QTL on b08 (*Yld*8.1) both under drought stress environments. Meanwhile under nonstress conditions the SPAD chlorophyll meter reading QTL on b06 accounted for up to 42.2% of variance whereas the stem TNC QTL on b05 explained 17.6%. QTL for stem TNC on b06 was intermediate in its contribution genetic variance (11.6%) whereas all remaining QTL were of low significance as measured by their effect on genetic variance under drought or non-stress environments. Our study was based on very diverse sites with different rainfall patterns and photoperiods, which may have influenced the consistency of drought tolerance measurements across sites. It was notable that all the QTL except that for grain yield and stem TNC on b06 had a lower proportion of genetic variance accounted for by the full QTL model under drought stress environments than under non-stress environments.

Validation of the QTL from the mixed-model analysis was realized with a single-environment QTL analysis for each trait using composite interval mapping. This resulted in a much larger number of QTL than in the mixed-model analysis as would be expected. A total of 69 composite interval mapping QTL were detected (Table 6) across the four sites and across the nonstress and stress treatments in each environment. The majority of QTL (47 in total) were found in Palmira, given that for technical reasons all the traits were measured in this environment whereas in the African sites fewer traits were measured (Table 1). The second largest number of QTL was found for the Awassa site (18 in total) because this site also conducted the majority of the physiological measurements. In Malawi QTL were found for yield under drought stress and for SPAD chlorophyll meter readings. Interestingly, SPAD chlorophyll meter readings, which were measured in all the sites, had a similar number of composite interval mapping QTL for Awassa (n = 3) and Malawi (n = 4) and the greatest number of QTL (n= 6) detected in Palmira. No QTL were found for this trait in the drought-stress measurements in Amaro and variability for SCMR response in drought-stress and nonstress environments was observed. When comparing the QTL found under drought stress environments to those under nonstress environments, we found more in the former environments than in the latter environments.

**Table 6:** Significant QTL and the percentage of genetic variance explained by the QTL detected with composite interval mapping in the DOR364 × BAT477 mapping population grown under drought stress and nonstress conditions at four different locations in three countries (Colombia, Ethiopia, and Malawi) for photosynthate acquisition, accumulation, and partitioning traits

Trait	QTL Name*^a^*	LG	Marker	Additivity	Source	LR	R2	TR2
Yield	Yld_PALDS	7	BM210	48.22	BAT477	13.35	0.14	0.45
		8	P103	45.66	BAT477	15.48	0.12	0.37
	Yld_AMDS	4	U1002	54.01	BAT477	9.40	0.09	0.27
		8	P103	70.48	BAT477	11.63	0.11	0.25
	Yld_AMNS	1	BM200	169.91	BAT477	11.17	0.11	0.21
		8	O2002	222.84	BAT477	11.25	0.13	0.23
	Yld_KASDS	4	U1302	53.06	BAT477	11.12	0.11	0.21
		8	A402	55.46	BAT477	11.25	0.13	0.23
Canopy biomass	Cbm_PALDS	6	BMc238	215.65	DOR364	24.89	0.29	0.47
		10	P401	140.60	DOR364	17.42	0.14	0.38
	Cbm_PALNS	6	AB1001	312.66	DOR364	17.45	0.17	0.34
		10	N601	359.10	DOR364	19.03	0.16	0.33
		10	AI1403	295.66	BAT477	13.22	0.11	0.33
Pod harvest index	Phi_PALDS	3	Q1001	0.81	BAT477	17.87	0.17	0.41
		4	AI1402	0.63	BAT477	13.27	0.11	0.38
		8	O1603	0.58	BAT477	11.14	0.09	0.37
		8	BM153	0.57	BAT477	10.15	0.08	0.37
	Phi_PALNS	1	BMc224	0.78	BAT477	21.79	0.26	0.46
		6	BMc238	0.92	BAT477	18.33	0.33	0.58
	Phi_AWNS	1	BM200	1.52	BAT477	14.94	0.12	0.39
		5	P102	1.92	BAT477	15.67	0.12	0.38
		6	Y501	1.28	BAT477	11.71	0.09	0.38
Pod partitioning index	Ppi_PALDS	2	AB1003	3.70	BAT477	10.29	0.08	0.37
		2	BMa33	5.69	DOR364	21.32	0.18	0.36
		5	P101	2.92	BAT477	9.53	0.07	0.36
		11	AB502	4.10	BAT477	13.15	0.15	0.42
	Ppi_PALNS	2	F603	2.92	DOR364	9.91	0.10	0.40
		10	GATs54	5.14	BAT477	27.02	0.23	0.38
	Ppi_AWDS	4	AI1401	4.44	DOR364	12.84	0.11	0.35
		8	M1002	5.49	BAT477	10.22	0.09	0.28
		9	Y1701	5.15	BAT477	9.46	0.15	0.45
	Ppi_AWNS	9	BMc255	6.99	BAT477	12.63	0.14	0.04
Stem biomass reduction	Sbr_PALDS	4	BMa12	3.29	BAT477	12.57	0.11	0.29
	Sbr_PALNS	1	BMc224	4.27	BAT477	12.66	0.14	0.39
		1	BMc313	5.26	DOR364	15.44	0.21	0.46
		6	Q401	5.55	DOR364	18.06	0.25	0.48
	Sbr_AWNS	1	BMc232	5.27	BAT477	16.56	0.14	0.36
Harvest index	Hri_PALDS	2	P701	5.05	DOR364	17.95	0.15	0.36
		5	P101	3.70	BAT477	15.59	0.13	0.36
		11	AB502	3.12	BAT477	10.51	0.09	0.37
	Hri_PALNS	10	U1401	4.34	BAT477	24.27	0.23	0.41
		10	Y801	3.21	DOR364	14.83	0.12	0.38
	Hri_AWDS	4	AI1401	3.66	DOR364	16.41	0.14	0.34
	Hri_AWNS	6	Y501	5.14	BAT477	10.86	0.11	0.32
		9	BMc292	4.78	BAT477	10.46	0.11	0.31
Stem TNC	Stc_PALDS	3	X1901	10.82	BAT477	11.18	0.09	0.36
		5	F601	10.03	BAT477	9.28	0.07	0.36
		6	M501	13.57	DOR364	16.0	0.14	0.30
	Stc_PALNS	5	F601	21.88	BAT477	29.90	0.26	0.40
Seed TNC	Snc_PALDS	2	Q1401	15.52	BAT477	11.02	0.10	0.27
	Snc_PALNS	9	BMc255	13.70	BAT477	15.43	0.15	0.33
SCMR	Scr_PALDS	2	P1602	0.85	BAT477	10.25	0.07	0.42
		6	BMc238	1.23	DOR364	19.90	0.15	0.42
		8	P103	0.87	BAT477	10.44	0.07	0.42
	Scr_PALNS	3	AE103	1.63	BAT477	15.14	0.08	0.59
		4	O701	2.22	DOR364	17.40	0.14	0.67
		6	BMc238	3.56	DOR364	55.44	0.37	0.59
	Scr_AWDS	3	X901	0.87	BAT477	10.33	0.10	0.34
		10	BMc66	0.82	BAT477	10.61	0.09	0.32
	Scr_AWNS	4	BMa288	0.97	BAT477	10.51	0.11	0.35
	Scr_KASDS	8	AC702	0.86	BAT477	10.18	0.09	0.31
	Scr_KASNS	4	BMa20	1.03	BAT477	12.95	0.12	0.43
		6	BMc238	1.0	DOR364	13.46	0.10	0.43
		10	M902	1.35	DOR364	17.34	0.13	0.43
Leaf area index	Lai_PALDS	4	S1301	0.15	DOR364	15.23	0.13	0.35
		7	AF1902	0.16	BAT477	16.44	0.14	0.39
	Lai_PALNS	4	Pv-a1001	0.12	DOR364	9.40	0.08	0.32
		8	A402	0.20	DOR364	19.58	0.15	0.41
		8	P103	0.16	BAT477	13.40	0.10	0.38

QTL, quantitative trait loci; LG, linkage group; LR, likelihood ratio; TNC, total nonstructural carbohydrate content; SCMR, SPAD chlorophyll meter reading.

a QTL trait code (first three letters) as for Table 5 and QTL association with specific environments following abbreviations as given in Table 4.

The percentage phenotypic variance explained by individual, site-specific QTL varied from 7% to 37%, with the greatest values found for the SCMR trait and lowest values for yield, leaf area index, and seed TNC. Stem TNC had R2 values up to 0.26, which was similar to stem biomass reduction (up to 0.25), harvest index (0.23), and canopy biomass (0.29). Pod harvest index QTL had greater variances explained (up to 0.33) than pod partitioning index (up to 0.23).

## Discussion

### G×E interactions for grain yield

Drought is a major constraint contributing to yield reduction in common bean production. In this study, drought stress treatments caused an average of 62% yield reduction relative to the nonstress environments. The effect varied from location to location but was always negative, resulting in yield loss. The greatest drought stress occurred during late planting environments of Awassa and Amaro as compared with Kasinthula and Palmira rain-fed environments. This may have been also attributable to the combined effect of bean stem maggot and drought on plant establishment and yield. As a result, ranges in yield among RILs were large in Amaro and Awassa as compared with Kasinthula and Palmira (Table 2). A large yield variation among locations indicated that the drought stress environments used in this study were diverse, and hence cross-environment selection would minimize selection efficiency whereas yield selection is a trait that should be evaluated on a per-site basis.

All trial environments contributed considerably to the G×E variance of the traits considered in this study, and one cannot pool the African sites *vs.* the Latin American site for environmental effects (Figure 3). The magnitude of G×E variances was larger than that of the genotypic variance for all traits except for that of leaf area index (Table 3), indicating there were sizeable differences in genotypic responses of RILs across environments for the vast majority of traits. Furthermore, large dissimilarities among test environments were observed to be the cause of high variance among genotypes (Table 4, Figure 3). The genetic correlations for a given trait between trial environments, therefore, were generally low. This was especially the case for grain yield performance. Some exceptions were found for the same trait in the same location within African sites but overall the results indicated the lack of an ideal and representative test environment in discriminating the potential of test genotypes for reliable inference to be made on the performance across all other environments.

### Breaking down grain yield into component traits

Understanding factors that account for larger differences in achieved yield compared with potential yield and the genetic enhancement for characters that contribute to yield formation are prime targets for physiology or molecular-aided approaches to crop improvement. Yield is a constant capacity system and a result of often interdependent traits (Yan and Wallace 1995). Two processes: namely, carbon assimilation rate and proportion of assimilates allocated to the storage organs, play an important role in determining achieved yield and yield potential of a crop or its varieties (Blum 1998). Carbon assimilation depends on sustained photosynthetic ability of the source whereas sink strength determines the ability of the storage organ to import and use the available assimilate. Meanwhile, photosynthate remobilization between source and sink are especially important for legumes, which often remain green-stemmed at the end of the season and which therefore are poor at removing carbohydrates from roots and stems to grain.

The present study accounts for genetic and environmental variation in grain yield of common bean by assessing the contribution of different traits to the three aforementioned processes described, which can be summed up as: photosynthetic ability, photosynthate accumulation, and photosynthate partitioning. Traits assessed that contributed to plant photosynthetic ability included leaf area index, leaf chlorophyll content (assessed as SPAD reading), and canopy temperature depression. Canopy biomass production and stem and seed TNC were used to assess photosynthate accumulation, whereas partitioning of photosynthates were assessed using pod harvest, pod partitioning, stem biomass reduction, and harvest indices.

### Impact of factors contributing to photosynthate acquisition and photosynthetic ability

Among the photosynthate acquisition, accumulation, and partitioning traits, the proportion of variation in yield accounted for by variation in traits that could potentially contribute to plant photosynthetic ability under drought stress was variable. For example, leaf area index had a direct increasing effect on yield under both drought stress and nonstress environments (Table 3). Meanwhile, other traits had no similar direct increasing effect on yield. The explanation for the importance of leaf area index could be in that leaf area reduction by inhibition of new leaf growth or via the earlier senescence of older leaves would lead to decreased transpirational area. However, a smaller leaf area index and less number of leaves could also result in lower intercepted radiation throughout the growth season and ultimately decreased biomass production (Pereira and Chaves 1993).

The positive and significant correlations of leaf area index with grain yield both under drought stress and nonstress environments in this study indicated that genotypes with maximum possible leaf area produced greater yield. SPAD cholorophyll meter reading and canopy temperature depression showed slight increases in response to drought in both parents and RILs whereas that of leaf area index was decreased (Table 2). The genetic variance was about equal to the G×E variance for these traits, indicating relatively high across environment repeatability (Table 4).

Despite this, SPAD chlorophyll meter reading showed inconsistent association with grain yield both under stress and nonstress environments whereas the correlations of grain yield with canopy temperature depression were not significantly different from zero. Slight increases in SPAD chlorophyll meter reading and canopy temperature depression during drought stress (Table 2) might suggest maintaining greener leaves and cooler canopy temperature would contribute to higher photosynthetic ability for sustained grain filling during stress or greater absorption and use of water during the growing period.

However, from our results it was difficult to make conclusions about the entire growth period as SPAD and canopy temperature readings were measured in single time points during this study. Therefore, the lack of well-structured correlations with grain yield may be as much a function of timing of measurements as physiological combinations of processes affecting both leaf chlorophyll and canopy temperature or water content during development.

### Impact of factors contributing to photosynthate accumulation

Among the traits having to do with photosynthate accumulation, the proportion of variation in yield accounted for by variation in canopy biomass dry weight at mid-pod filling was significant. Canopy biomass dry weight at that stage had a direct positive effect on yield both under drought stress and non-stress environments across locations. Improved canopy biomass production, therefore might have contributed to drought tolerance. However, its utility as a trait for indirect selection for drought tolerance is questionable because it is not easy to measure, is destructive and also has a large environmental effect on its expression.

Another study performed by Ramirez-Vallejo and Kelly (1998) indicated that canopy biomass had a strong association with stem diameter, which may be easier to measure and is certainly a better trait in being nondestructive. But our personal experiences from field observations showed that genotypes with strong and thick stems were not always good yielding materials under drought stress.

Moreover, negative and nonsignificant correlation of stem TNC with grain yield indicated that the postflowering drought stress in this study did not enhance stem photosynthate source remobilization to the seed in the RIL population. Alternatively, stem TNC remobilization did not increase seed weight and per plant yield. This finding suggests the need to study the importance of stay green stems and delayed leaf senescence in common bean as an immediately-available source of photosynthates for remobilization to reproductive parts that might provide a longer window for better grain filling under drought stress. Blum (1998) practiced selection for stem reserves as potent trait to improve grain filling under drought stress in wheat.

On the other hand, stem and seed TNC had low or negative direct effect on grain yield both under drought and nonstress environments (Table 3). The effect of drought stress on biomass production was larger than that on grain yield, whereas the effect was moderate on stem TNC (Table 2). Apart from these observations, slight increases in average seed TNC were observed across the RILs under drought stress. However, the correlation of seed TNC with grain yield was negative and low under the drought environments, indicating that seed TNC increase is not a useful predictor of drought tolerance in common bean. Moreover, the genotype to G×E ratios for stem and seed TNC (0.81 and 0.71, respectively) and canopy biomass dry weight at mid-pod filling (0.55) were generally low indicating low across environment repeatability for these traits (Table 3).

### Impact of factors contributing to photosynthate partitioning

Yield improvements via photosynthate partitioning traits were also affected by drought stress but not as much as the photosynthate ability and accumulation related traits. In general, partitioning traits were positively associated with final grain yield under drought stress except for pod harvest index in the severe drought stress environment of Awassa.

Positive associations of partitioning indices with grain yield under drought stress suggest the importance of photosynthate remobilization from different vegetative structures of plant to contribute to increased seed weight. Data supporting similar relationships of yield with photosynthate partitioning indices under drought have been reported (Ramirez-Vallejo and Kelly 1998). However, negative correlation of pod harvest index with grain yield in the severe drought stress environment at Awassa indicates that this trait may not be useful as an indirect selection tool for all drought conditions.

Mobilization of photosynthates from pod wall reserve to final grain might be impaired by thicker pod wall formation under severe drought stress condition as compared with moderate drought stress conditions. Under moderate drought stress in Palmira, Colombia, pod harvest index had positive and significant correlation with grain yield but not in the more severe stress of Awassa, Ethiopia.

Finally, the partitioning traits particularly pod partitioning index, stem biomass reduction, and harvest index had low associations with grain yield under nonstress environments. This could have been due to the indeterminate growth habit of the population, especially as some of the RILs continued to produce pods along with late rain showers even after the destructive sampling of mid-pod fill used to calculate these indices (Figure 1). For the partitioning traits, even if no parental difference was observed, transgressive segregation among the RILs was important, making these traits of potential interest in further populations for drought improvement in common bean.

Our results showed that improved remobilization of photosynthate to grain under drought condition may be an important mechanism to enhance yield formation under some conditions but also suggest that pyramiding of various tolerance mechanisms might be needed for breeders to improve drought adaptation in common bean. The best-bet mechanisms would be to pyramid deeper rooting that contribute to greater water uptake, improved photosynthetic ability via maximum possible leaf area for radiation interception and ability to control stomatal opening for better photosynthate accumulation along with better remobilization to grain under drought stress.

### Improving our genetic understanding of drought tolerance traits

This study amplified our understanding of the mechanisms and genetics of drought tolerance. Our principal achievement was to analyze three different global categories of traits related with the processes contributing to yield formation in common bean under drought, namely photosynthetic ability, photosynthate accumulation, and photosynthate remobilization to grain. Specifically, we identified the best DNA markers linked to these traits across different drought stress and non-stress environments. Harvest index, which reflects the differences in the photosynthate partitioning process, was not easy to quantify in common bean because of leaf fall during pod filling and this was further analyzed through three indices: pod partitioning index, stem biomass reduction, and pod harvest index. The stable QTL identified in this study, although few in number compared with the site-specific QTL (Tables 5 and 6), would be the most useful to harness for indirect selection in breeding.

For example, the QTL for leaf area index and leaf chlorophyll content, canopy biomass, stem TNC, pod harvest, pod partitioning, stem biomass reduction, and harvest index all appear to improve drought adaptation across various environments in common bean. Meanwhile, the QTL for yield *per se* under drought stress would be interesting to select for as one overlapping QTL was found for yield for each site (Table 6).

Multienvironment mixed model mapping in the RIL population showed that QTL for the above mentioned traits were mostly distributed on linkage groups b03, b05, b06, b08, b09, and b10 (Table 5, Figure 4). For the traits with significant QTL, mostly one QTL was detected, highlighting the difficulty in detecting major QTL in drought studies for common bean under managed stress conditions in the field. A larger RIL population could identify more minor QTL but would be difficult to manage under the precision of replicated lattice design experiments used in our study. Notably, we found an important mixed model QTL for grain yield (*Yld*8.1) across various drought stress environments that was associated with the marker P103 on linkage group b08. This QTL was also detected in single-environment CIM analysis for Amaro, Kasinthula, and Palmira and therefore is consistent and worth selecting for using marker assisted selection.

Composite interval mapping found a large number of QTL for other traits (4 for canopy biomass, 9 for pod harvest index, 10 for pod partitioning index, 6 for stem biomass reduction, 7 for harvest index, 6 for stem or seed total nonstructural carbohydrates, and 13 for SPAD chlorophyll meter readings). However, many of these did not fit with results from QTL detection under the multienvironment mixed model, which may indicate that the mixed-model approach is not useful when there is significant G×E interaction detected for a trait or when opposing effects are noted in the biplot analysis. For example, the pod partitioning index, grain harvest index, and pod harvest index mixed-model QTL of *Ppi*3.1, *Hri*3.1, and *Phi*6.1 were not consistent with results from single-environment analyses of these same traits. All of these traits showed poor associations across environments in the biplot analyses and had the lowest of all heritabilities observed among the traits.

Meanwhile, the *Scr*6.1 QTL locus from the mixed-model analysis was associated with two single-environment QTL from the CIM analysis, namely those for Kasinthula and Palmira. Notably, SPAD chlorophyll meter readings, which was the trait associated with this QTL, were similar for each of the environments in the biplot analysis and had intermediate heritability. Because SPAD chlorophyll meter readings were not associated with yield, we expected the QTL for these traits to be independent, and they were, except that some single-environment QTL were found on linkage groups b03, b04, b08, and b10. Finally, the mixed-model QTL for *Stc*5.1 and *Stc*6.1 aligned with the single-environment QTL, but this was based on only analysis in Palmira for stem TNC so this was to be expected.

Interestingly, the CIM single-environment analysis found more QTL under drought stress than under nonstress conditions. This may be due to more substantial and significant phenotypic differences between RIL lines in the drought stress environments than in the nonstress environments. This was to be expected, given the differences between the mapping population parents, which differ under drought stress for most traits compared with nonstress environments. Supporting this conclusion, the mixed-model QTL for canopy biomass (*Cbm*3.1), leaf area index (*Lai*10.1), and stem biomass reduction (*Sbr*9.1) did not align with QTL from the CIM analysis perhaps, suggesting that the mixed model approach is only useful when analyzing data from various similar environments rather than from the contrasting environments of drought stress and nonstress.

In previous studies with composite interval mapping in this and another RIL population, authors found small numbers of QTL for yield under drought (Schneider *et al.* 1997; Blair *et al.* 2010). Therefore, the identification of yield QTL on linkage group b08 that is stable across environments is a notable achievement. The fact that the positive allele for yield at this QTL is for the most part BAT477 but sometimes DOR364 indicates that one must consider the effect of substituting alleles when breeding for multiple environments. Apart from this, yield QTL were not linked with or pleiotropic to biomass accumulation and photosynthate remobilization traits, even though positive phenotypic correlations with some of these traits existed. This finding indicated that different sets of genes at different regions of the genome are activated in the yield accumulation process. This is attributable to the nature of yield as a complex trait determined by many physiological processes during growth and development.

Because remobilization of photosynthates from vegetative plant structures to pod wall and from pod wall to the final grain yield is an important mechanism in drought adaptation for common bean, it is interesting to see where QTL for these traits were located and to assess any possible functional relationship with other traits. In this regard, QTL for traits related with photosynthate accumulation and partitioning were distributed on linkage group b03, b05, b06, and b09 with genetic pleiotropy on b03 for pod partitioning and pod harvest indices (Figure 4). Meanwhile, the QTL for SPAD chlorophyll meter reading or SCMR in the multienvironment analysis was located on the same linkage group (b06) with pod harvest index, and both traits had similar pattern of association with grain yield particularly under stress.

This may indicate that linkage group b06 contains genes for increased plant photosynthetic carbon assimilation per unit leaf area under moderate drought stress and perhaps a larger number of chloroplasts per cell in leaf tissues and/or greater activation status of the plant enzyme Rubisco. These genes may result in a larger pool of photosynthates for remobilization to the pod wall and then to the grain, ultimately resulting in increased yield. In the CIM analysis, many of the individual environment QTL were also detected on linkage group b06, but additionally QTL were found on linkage groups b02, b03, b04, b08, and b10, indicating that this may be a complex trait but one that is tractable to further genetic analysis given its high heritability and ease with which QTL are detected. The largest number of QTL for any given trait across the entire study was found for this trait, making it an interesting objective for QTL fine mapping and for map-based cloning or candidate gene analysis.

However, one must take into account that SPAD readings and QTL for this trait may be less useful under severe drought stress compared with moderate stress. This would be because very thick, small, and dark green leaves under drought might be less photosynthetically active because of closed stomata despite high chlorophyll content. Leaves of common bean during severe drought became very dark green as compared with moderate-stress or nonstress environments as reflected in greater SPAD meter reading (Table 2). Thick leaves would be expected to have more chloroplasts but construction and maintenance of thicker leaves is costly in terms of carbon (Lambers *et al.* 2008). Hence, thicker leaves might have no advantage to productivity during severe drought stress in common bean but rather reflect a structural adjustment to the photosynthetic apparatus while conserving water as a survival strategy. Increased leaf thickness or reduced specific leaf area reflects a decreased cell expansion under drought stress.

Finally, the negative correlation of pod harvest index with final grain yield under severe drought stress indicated a major effect of severe drought on allocation of photosynthate from the pod wall to final grain production as reflected by seed weight. Differences in QTL location were observed for SPAD chlorophyll meter reading and stem TNC in pre-flowering, greenhouse-grown plants subject to terminal stress (Asfaw and Blair in press). Therefore, postflowering measurements of stem TNC and SPAD reading may give different results from preflowering measurements of these traits. As a result we may postulate a different set of genes activating under drought stress in these different stages of plant growth as the plant moves from vegetative to reproductive phases.

### QTL × environment interactions

The estimates of environment-specific QTL effects revealed that each site and season exerted a large effect on the expression of both yield and photosynthate remobilization or accumulation traits but less so on expression of photosynthate carbon assimilation traits. For example, it was notable that the QTL × environment interaction was of the cross-over type for grain yield and canopy biomass dry weight at mid-pod filling.

Meanwhile, the colocalizing QTL for pod partitioning index and harvest index indicating greater relationship between these traits across environments. These traits also had low-to-medium genotype to G×E interaction variance ratio, confirming greater influence of the environment on trait expression. The QTL × environment interactions indicate that site-specific mechanisms of drought tolerance are important. This makes sense from the perspective that no two drought events are the same and also that soil variability in terms of nutrients, porosity and structure strongly affect severity of drought effects.

Weather variability in terms of night and day temperatures can also be important characteristics of individual drought events. Plants usually express differential adaptive strategies to drought stress, which may not be mutually exclusive but in practice are rarely combined within agronomically superior genotype (Ludlow 1989). For example, deep rooting or high levels of chlorophyll content alone does not assure yield formation under drought stress. Instead a range of adaptive responses to drought might be found in plants and mechanisms of drought tolerance would not necessarily be linked genetically or related physiologically.

For example, the QTL for grain yield and photosynthate remobilization traits did not overlap with any of the rooting depth or rooting pattern QTL detected for this population (Asfaw and Blair, in press). This indicates that different sets of genes and physiological mechanisms determine activation of rooting depth traits that allow extraction of water from a greater depth compared to genes and mechanisms that affect photosynthate accumulation and its remobilization for yield improvement under drought stress. Given this detailed analysis of multiple drought tolerance mechanisms in a single mapping population, geneticists and plant breeders can determine the importance of each individual mechanism for further studies and analyses.

### Conclusions

There are four major insights to be gained from this study: (1) when using composite interval mapping for each individual environment many QTL are detected but these tend to be site-specific; (2) meanwhile, when using a multienvironment mixed-model approach, only a few major QTL were detected for traits measured in this study due to strict consideration of G×E interaction which is important for some low heritability traits; (3) high QTL × environment interaction for the significant QTL was found except for the loci *Stc6.1*, *Sbr9.1*, and *Lai10.1* (Figure 4); and (4) low total genetic variance was explained by the QTL.

The low number of stable QTL, the high QTL × environment interaction and the large proportion of genetic variance unexplained by the individual mixed-model QTL highlight the difficulty in detecting QTL in drought studies under field stress. The QTL tagged in this study for traits related with photosynthate remobilization will be useful in common bean breeding programs aimed at improving yield potential in stressful environments since phenotypic selection for such physiological traits is destructive and laborious. Furthermore, the results from our work will permit genetic studies to focus on certain parts of the genome and certain physiological processes that improve photosynthate acquisition, accumulation or remobilization for yield improvement under drought stress. Marker-aided selection can pave the way to breed new varieties of drought tolerant common bean that combine a range of tolerance mechanisms in commercial grain types.
